# PI3Kδ inhibition causes feedback activation of PI3Kα in the ABC subtype of diffuse large B-cell lymphoma

**DOI:** 10.18632/oncotarget.20864

**Published:** 2017-09-13

**Authors:** Georgios N. Pongas, Christina M. Annunziata, Louis M. Staudt

**Affiliations:** ^1^ Lymphoid Malignancies Branch, National Cancer Institute, National Institutes of Health, Bethesda, MD, USA; ^2^ Medical Oncology Service, National Cancer Institute, National Institutes of Health, Bethesda, MD, USA; ^3^ Women’s Malignancies Branch, National Cancer Institute, National Institutes of Health, Bethesda, MD, USA

**Keywords:** DLBCL, PI3K, Idelalisib, ABC DLBCL, BYL719

## Abstract

Cell line models of the activated B cell-like (ABC) subtype of diffuse large B cell (DLBCL) depend on both NF-κB and phosphatidylinositol 3-kinase (PI3K) signaling pathways for survival, especially those with gain-of-function B cell receptor (BCR) mutations. Here we show that these cells depend specifically on the PI3Kδ isoform, but that PI3K pathway interruption by PI3Kδ inhibitors is short-lived due to feedback activation of the PI3Kα isoform. PI3Kδ and PI3Kα inhibition cooperated in killing ABC DLBCL lines, and genetic knockdown of PI3Kα sensitized cells to PI3Kδ inhibition and prolonged the interruption of PI3K signaling. PI3Kδ inhibition evoked feedback activation of proximal BCR signaling, which increased the association of PI3Kα with BCAP and CD19 and increased overall PI3K activity. These results support the clinical evaluation of dual PI3Kδ and PI3Kα inhibition in patients with ABC DLBCL.

## INTRODUCTION

Diffuse large B cell lymphoma (DLBCL) is the most common B cell malignancy in the adult population. Gene expression profiling studies defined three types of DLBCL based on cell of origin (COO): the activated B cell-like (ABC) DLBCL, germinal center B cell-like (GCB) DLBCL and primary mediastinal B cell lymphoma (PMBL) [1, 2]. This taxonomy has clinical significance since ABC DLBCL has an inferior prognosis relative to other DLBCLs [3]. RNA interference screening and sequencing studies revealed that ABC DLBCL tumors are addicted to NF-κB pathway activity for survival [4-6]. One recurrent mechanism to activate NF-κB in ABC DLBCL is chronic active B cell receptor (BCR) signaling [4], which is initiated by self-antigen reactivity of the BCR and is augmented by gain-of-function mutations in the BCR subunits CD79A and CD79B in ∼21% of cases [4, 7]. A second mechanism to activate NF-κB involves gain-of-function mutations targeting MYD88, with one point mutation, L265P, occurring in 29% of cases [5]. In addition to NF-κB, chronic active BCR signaling in ABC DLBCL activates phosphatidylinositol 3-kinase (PI3K), which contributes to NF-κB activation in ABC DLBCL lines [4, 8].

PI3Ks are a family of kinases that regulate cell proliferation, metabolism, protein synthesis and survival [9]. Sensitivity to small molecules that inhibit all class I PI3Ks demonstrated a key role of PI3K in cell viability in a subset of ABC DLBCL lines with CD79B mutations and in GCB DLBCL lines with PTEN loss [10, 11]. Given the on-target toxicities of pan-PI3K inhibitors, clinical trials have been recently investigating isoform-specific PI3K inhibitors in solid tumors and hematologic malignancies. Idelalisib (CAL-101), a PI3Kδ-selective inhibitor, was the first PI3K inhibitor approved by the FDA for the treatment of relapsed or refractory CLL and indolent lymphomas [12, 13].

In this study we investigated the dependency of ABC DLBCL cell lines on particular PI3K isoforms and studied resistance mechanisms to isoform-specific PI3K inhibitors.

## RESULTS

### PI3Kδ inhibitors decrease PI3K activity in BCR-dependent ABC DLBCL lines

**Figure 1:**
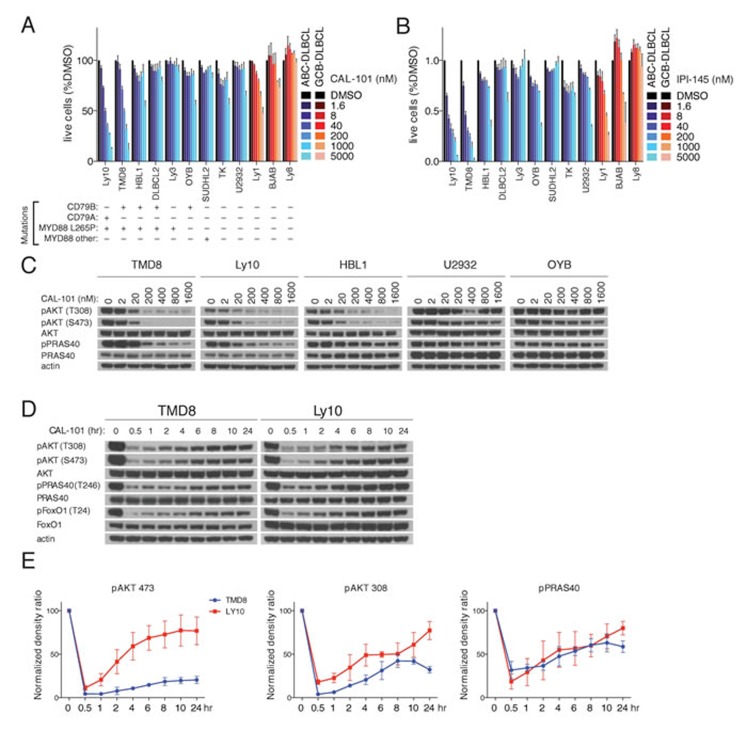
PI3K activity rebounds shortly after PI3Kδ inhibition in a subset of ABC DLBCL lines **A.**-**B.** MTS-cytotoxicity assay was performed in 9 ABC DLBCL and 3 GCB-DLBCL cell lines following 4 days treatment with CAL-101 or IP-I145 at the indicated concentrations. Data shown represent the mean ± SE of three independent experiments. **C.** The indicated DLBCL cell lines were exposed for 2hr to CAL-101 followed by cell lysis with non-denaturing lysis buffer. Western blot indicates that the PI3K activity of cell lines with MYD88 L265P and CD79A or CD79B mutations is regulated by the PI3Kδ isoform. **D.** TMD8 and LY10 were exposed over different time periods to CAL-101. Western blot indicates that PI3K activity rebounds shortly after PI3Kδ inhibition. Data shown is representative from at least three independent experiments. **E.** Densitometry of the biological triplicate experiment of panel 1D. Error Bars represent the SE of the densitometry.

A panel of 9 ABC DLBCL lines and 3 GCB lines were treated with the PI3Kδ-specific inhibitor CAL-101. Exposure to CAL-101 reduced the viability of 2 ABC DLBCL lines (Ly10, TMD8) that have gain-of-function mutations targeting the BCR subunits CD79A (Ly10) or CD79B (TMD8) as well as the MYD88 L265P mutation (Figure 1A). Similar results were obtained with IPI-145 (Duvelisib), a dual PI3Kγ,δ inhibitor, suggesting that Ly10 and TMD8 are dependent on the PI3Kδ isoform (Figure 1B).

To validate that the action of CAL-101 was on-target, PI3K activity was determined in a panel of BCR-dependent ABC DLBCL lines exposed to a range of CAL-101 concentrations, using AKT phosphorylation at threonine 308 (pAKT-308) and serine 473 (pAKT-473) as readouts. Near complete reduction of pAKT-308 and pAKT-473 levels was noted with 200 nM CAL-101 in three ABC DLBCL lines (TMD8, HBL1, Ly10) with concurrent BCR (CD79A or CD79B) and MYD88 (L265P) mutations, whereas CAL-101 at high concentrations (1600 nM) had little or no effect on PI3K activity in other ABC DLBCL lines (Figure 1C). Another indicator of PI3K signaling is phosphorylation of a substrate of AKT, PRAS40, at threonine 246 (pPRAS40) (Figure 1C). CAL-101 decreased pPRAS40 levels in TMD8 and Ly10 cells in a dose-dependent manner, suggesting engagement of downstream elements of the PI3K pathway.

### PI3Kα mediates early adaptive responses after PI3Kδ inhibition

Next we questioned whether prolonged treatment with a PI3Kδ inhibitor would increase its biological effects on ABC DLBCL cells. We analyzed TMD8 and Ly10 cells at various time points after CAL-101 treatment and unexpectedly observed a rapid rebound of pAKT-308, pAKT-473 and pPRAS40 levels within one 1-2 hr, suggesting the existence of a feedback mechanism to maintain PI3K signaling (Figure 1D-1E).

**Figure 2:**
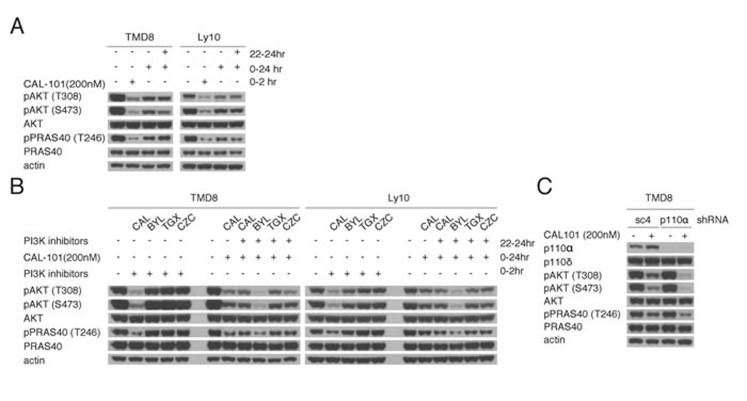
Reactivation of PI3K signaling following PI3Kδ inhibition is mediated through PI3Kα **A.** Pretreated TMD8 and Ly10 for 22hr with 200nM CAL-101 were re-challenged for 2hr with 200nM CAL-101. Western blot indicates that the rebound PI3K activity is resistant to CAL-101 re-challenge. Data shown is representative from at least three independent experiments. **B.** Left half of each blot: TMD8 and Ly10 were treated with BYL719 (150nM), CAL-101(200nM), TGX221(100nM) and CZC24832 (500nM) and harvested at 2hr. Right half of each half: TMD8 and Ly10 were treated with CAL-101(200nM) at time 0 and with BYL719 (150nM), CAL-101(200nM), TGX221(100nM) and CZC24832 (500nM) at 22hr and harvested at 24hr. Cells were lysed with non-denaturing lysis buffer. Western blot indicates that the rebound PI3K activity after CAL-101 treatment is sensitive to PI3Kα inhibition. Data shown is representative from at least three independent experiments. **C.** TMD8 cells were transduced with sc4 (control) or PI3Kα-targeted shRNA for 24hr, followed by 24hr CAL-101 treatment. Results indicate that the knock-down of PI3Kα prolongs the PI3K inhibition of CAL-101 without decreasing the baseline PI3K activity. Data shown is representative from at least three independent experiments.

One explanation for the rebound in PI3K activity after CAL-101 treatment could be a decrease in the concentration of active drug at later time points. To address this possibility, TMD8 and Ly10 cells were re-challenged at 22 hr with a second aliquot of CAL-101 (200 nM). Despite the addition of fresh drug, pAKT-308, pAKT-473 and pPRAS40 levels were unchanged, suggesting that the PI3K reactivation could be due to activation of a PI3K isoform other than PI3Kδ (Figure 2A). To investigate this possibility, we measured the expression of all four Class I PI3K isoforms as well as the regulatory p85 subunit by immunoblot and observed a modest increase in the level of the PI3Kα isoform after 6 hours of CAL-101 treatment of TMD8 cells, but this was not observed in Ly10 (Supplemental Figure 1A).

We used pharmacologic and genetic approaches to functionally evaluate the contribution of various PI3K isoforms to the rebound of PI3K activity following CAL-101 treatment. One approach utilized small molecule inhibitors that are selective for PI3Kα (BYL719), PI3Kβ (TGX221), and PI3Kγ (CZC24832) [14-16]. By dose titration, we determined that BYL719 (200 nM), TGX221 (100nM), and CZC24832 (500 nM) had no effect on PI3K activity as single agents after 2 hour treatment of TMD8 cells (Supplemental Figure 1B). Next, we treated TMD8 and Ly10 cells for 22hr with 200nM CAL-101 and then added the isoform-selective inhibitors for 2 hours at concentrations determined to have no effect on PI3K activity in untreated cells. Notably, the rebound increases in pAKT-308, pAKT-473 and pPRAS40 levels following CAL-101 treatment were abrogated by treatment with the PI3Kα inhibitor BYL719, but not by treatment with the PI3Kβ and PI3Kγ inhibitors or by retreatment with CAL-101 (Figure 2B).

To verify the feedback role of PI3Kα genetically, we knocked down the expression of PI3Kα using a small hairpin RNA (shRNA) in TMD8 and Ly10 cells. Knockdown of PI3Kα did not decrease baseline PI3K activity, as measured by pAKT-308 and pAKT-473, and pPRAS40 (Figure 2C, Supplemental Figure 1C). Notably, following 24 hours of CAL-101 treatment, knockdown of PI3Kα did decrease the levels of these PI3K pathway indicators compared to cells transduced with a control shRNA (sc4, Figure 2B). Similar results were observed in HBL1, an ABC DLBCL line that showed some sensitivity to high concentrations of CAL101 and IPI-145 (Figure 1A-1B) and was shown previously to be sensitive to pan-PI3K inhibitors [8] (Supplemental Figure 1C). Together, these pharmacologic and genetic data suggest that PI3Kα is involved in the reactivation of PI3K signaling after PI3Kδ inhibition in ABC DLBCL cells.

### Cooperative effect of PI3Kα and PI3Kδ inhibition on viability and NF-κB activity in ABC DLBCL

**Figure 3:**
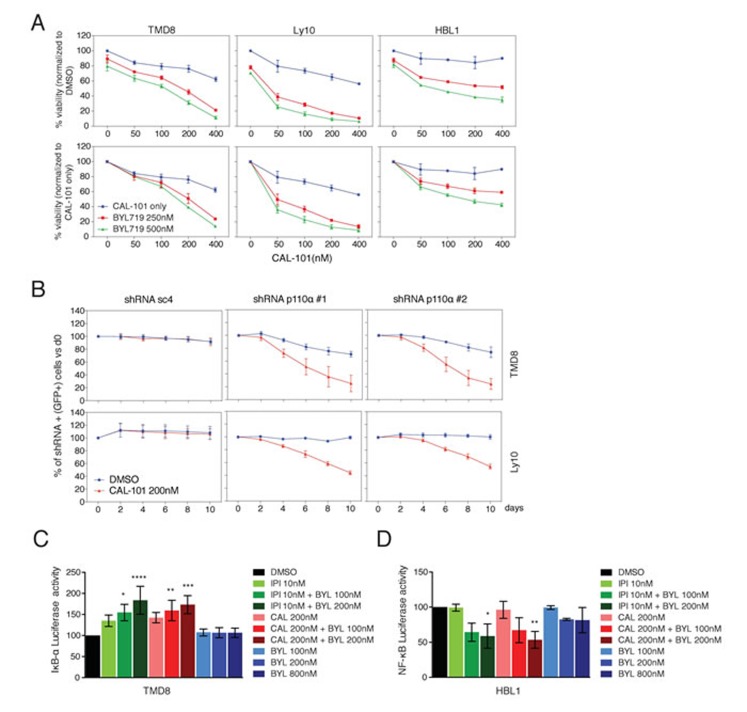
Combined PI3Kα and PI3Kδ inhibition cooperates to decrease viability and NF-κB activity in ABC DLBCL **A.** MTS-cytotoxicity assay was performed in TMD8 and Ly10 after combined CAL-101 and BYL719 treatment. Upper panels display data normalized to DMSO treated cells, whereas lower panels display data for the combination treatment after normalization for the toxicity of CAL-101 alone. Results indicate at least a super-additive effect with the combined treatment. Data shown represent the mean ± SD of two independent experiments. **B.** TMD8 and Ly10 were transduced with sc4 (control) and PI3Kα-targeted shRNA and the percent of GFP+/shRNA+ cells was tracked over time under DMSO *vs* CAL-101 treatment. Results indicate that knock-down of PI3Kα sensitizes cells to PI3Kδ inhibition. Data shown represent the mean ± SE of three independent experiments. **C.** Relative activity of an IKBα-dependent luciferase reporter in TMD8 treated overnight with the indicated PI3K inhibitors (CAL-101: PI3Kδ; IPI-145: dual PI3Kγ, δ; BYL719: PI3Kα). Data shown represent the mean ± SE of three independent experiments. *, *p* = 0.0148. ****, *p* < 0.0001. **, *p* = 0.0054. ***, = *p* = 0.0003 **D.** Relative activity of an NF-κB-dependent luciferase reporter in HBL1 treated overnight with the indicated PI3K inhibitors (CAL-101: PI3Kδ; IPI-145: dual PI3Kγ, δ; BYL719: PI3Kα). Data shown represent the mean ± SE of three independent experiments. *, *p* = 0.0260. **, *p* = 0.0065.

To address the functional consequences of combined PI3Kα and PI3Kδ inhibition, the cytotoxicity of BYL719 and CAL-101, alone and in combination, was assessed in TMD8, Ly10 and HBL1 cells (Figure 3A). The upper panels represent data normalized to DMSO-treated cells. In the lower panels, the effects of combination treatments were additionally normalized to the effects of CAL-101 treatment alone (blue lines), meaning that a left shift of the drug combination toxicity curves indicates more than additive toxicity. Clearly, the addition of BYL719 cooperated with CAL-101 in killing all three ABC DLBCL lines in a dose-dependent manner (Figure 3A).

We next took a genetic approach to investigate the cooperation of PI3Kα and PI3Kδ inhibition in killing TMD8 and Ly10 cells. In both lines, knockdown of PI3Kα using two different shRNAs sensitized cells to CAL-101 treatment, whereas a control shRNA (sc4) did not (Figure 3B).

Since, the BCR pathway is known to activate NF-κB signaling in ABC DLBCL, we investigated whether combined PI3Kα and PI3Kδ inhibition interferes with NF-κB activation using two complementary assays. One assay measures the activity of IκB kinase (IKK), which activates the classical NF-κB pathway by phosphorylating IκBα [17]. For this assay, cells were engineered to express a fusion protein between luciferase and IκBα, such that inhibition of IKK causes a rise in luciferase levels [17]. Treatment with CAL-101 or IPI-145 alone inhibited IKK activity, but BYL719 had no effect (Figure 3C). Addition of BYL719 to either PI3Kδ inhibitor inhibited IKK further in a dose-dependent manner, indicating synergism, given the ineffectiveness of BYL719 treatment alone. In a second assay for NF-κB activity, HBL1 cells were engineered to express a reporter in which luciferase expression is driven by an NF-κB-dependent promoter. Treatment of these cells with CAL-101, IPI-145 or BYL719 had no effect alone, but the addition of BYL719 to either PI3Kδ inhibitor decreased NF-κB activity in a dose-dependent manner (Figure 3D). These results suggest that both PI3Kα and PI3Kδ can contribute to NF-κB activation in ABC DLBCL and that combined inhibition of both isoforms cooperates in reducing NF-κB.

### Activation of PI3Kα is mediated through increased proximal BCR signaling

**Figure 4:**
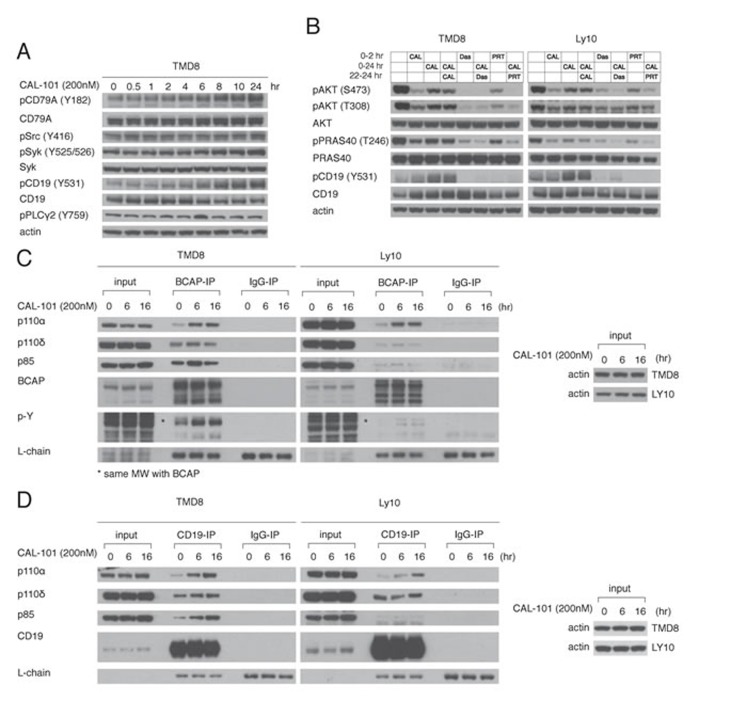
Feedback activation of PI3Kα following PI3Kδ inhibition depends on increased BCR signaling **A.** TMD8 was exposed over different time periods to CAL-101. Western blot indicates increased proximal BCR signaling following PI3Kδ inhibition. **B.** TMD8 and Ly10 were treated with 200nM CAL-101, 50nM Dasatinib (src inhibitor), 1000nM PRT062607 (Syk inhibitor) at the indicated time points and harvested at 2hr and 24hr. Results indicates that rebound PI3K reactivation following PI3Kδ inhibition is sensitive to Src and Syk inhibition. **C.** TMD8 and Ly10 were treated with CAL-101 over 0, 6 and 16hr. Cells were harvested, lysed with NP-40 lysis buffer, immunoprecipitated with BCAP and probed for the indicated proteins. **D.** TMD8 and Ly10 were treated with CAL-101 over 0, 6 and 16hr. Cells were harvested, lysed with NP-40 lysis buffer, immunoprecipitated with CD19 and probed for the indicated proteins.

The BCR is a major regulator of the PI3K activity in ABC DBLCL cells since knockdown of the BCR subunit CD79A profoundly decreases AKT phosphorylation [4]. Thus, we hypothesized that PI3Kα activation following PI3Kδ inhibition may be due to increased proximal BCR signaling. To this end, the phosphorylation of the BCR and proximal components of the BCR pathway was measured following PI3Kδ inhibition. In TMD8 cells, CAL-101 treatment (200 nM) led to phosphorylation of CD79A, and SYK, indicating increased proximal BCR signaling (Figure 4A).

To functionally investigate the contribution of proximal BCR signaling to PI3K regulation, we utilized the Src-family kinase inhibitor dasatinib and the Syk inhibitor PRT062607 (PRT). We identified concentrations of dasatinib (50nM) and PRT (1000nM) that inhibited baseline pAKT-308 and pAKT-473 levels in TMD8 and used these concentrations in subsequent experiments (Supplementary Figure 2A-2B). The rebound in PI3K activity that occurred after treatment with 200nM CAL-101 for 22hr was reversed by treatment for 2 hours with dasatinib or PRT in TMD8, Ly10 and HBL1 cells (Figure 4B, Supplementary Figure 2C), suggesting that feedback activation of PI3Kα is downstream of the BCR.

Since CD19 and BCAP participate in PI3K activation following BCR activation by recruiting PI3K through their YXXM motifs [18-20], we used immunoprecipitation to investigate the association of PI3K isoforms with these proteins at baseline and following 6hr or 16hr treatment of TMD8 and Ly10 cells with CAL-101 (200nM). At baseline, the PI3Kδ p110 subunit was associated with both CD19 and BCAP, as was the p85 regulatory subunit. While there was some association of the p110 subunit of PI3Kα at baseline with both CD19 and BCAP, its association increased over time of treatment with CAL-101 (Figure 4C, 4D). In addition, over the course of CAL-101 treatment, phosphorylation of BCAP (Figure 4C) and CD19 (Figure 4B) increased, which could contribute to PI3Kα recruitment. Together, our data point to an increase in proximal BCR signaling caused by PI3Kδ inhibition that contributes to the rebound in PI3K activity, at least in part by increasing the association of PI3Kα with BCAP and CD19.

## DISCUSSION

A subset of ABC DLBCL lines with BCR mutations are sensitive to pan-PI3K inhibitors [8, 11, 21]. Here, we show that PI3K activity in these ABC DLBCL lines is due to PI3Kδ, rendering them sensitive to the PI3Kδ-specific inhibitor CAL-101. We demonstrate that PI3K activity partially rebounds shortly after PI3Kδ inhibition and is resistant to CAL-101 re-challenge. With the utilization of isoform-specific inhibitors, we show that the rebound in PI3K activity is due to PI3Kα activation, a conclusion that was supported by shRNA-mediated knockdown of PI3Kα. The combination of PI3Kδ and PI3Kα inhibitors cooperated in killing ABC DLBCL lines, and PI3Kα knockdown sensitized ABC DLBCL lines to PI3Kδ inhibition. Finally, we show that compensatory proximal BCR signaling is evoked by PI3Kδ inhibition, leading to increased phosphorylation of BCAP and CD19 and subsequent recruitment of PI3Kα.

Our findings have clinical implications in ABC DLBCL and suggest that both PI3Kδ and PI3Kα would need to be pharmacologically targeted to achieve maximal clinical activity. A trial of the pan-PI3K inhibitor BKM-120 in relapsed/refractory non-Hodgkin lymphoma yielded a 12% objective response rate in DLBCL, but the subtype of the DLBCL tumors was not investigated [22]. Interestingly, copanlisib, a pan-PI3K inhibitor with preferential activity against PI3Kδ and PI3Kα, was recently reported to induce a partial response in one patient with relapsed/refractory DLBCL and the updated results of phase II trial are anticipitated [23, 24]. Our data suggest that dual PI3Kδ and PI3Kα inhibition should be evaluated clinically in ABC DLBCL, with molecular profiling of tumors to determine whether mutations in CD79A or CD79B are enriched in responding patients.

## MATERIALS AND METHODS

### Cell culture and drugs

The ABC DLBCL lines TMD8, HBL1, OYB, U2932, SUDHL2, and TK, and the GCB-DLBCL lines BJAB, OCI-Ly1, and OCI-Ly8 were grown in RPMI 1640 plus 20% FBS plus Pen/Strep. ABC DLBCL lines OCI-Ly10, OCI-Ly3 and DLBCL2 were grown in RPMI 1640 plus 20% heparinized human plasma plus Pen/Strep. Cell lines were previously engineered to express an ecotropic retroviral receptor and a bacterial tetracycline repressor [4]. All drugs (PRT062607, Dasatinib, BYL719, CAL-101, IPI-145, TGX221, CZC24832) were obtained from Selleckchem and diluted in DMSO.

### Cell viability (MTS) assay

For single-drug and drug combination viability assays, cells were plated in 96-well plates. Triplicate or duplicate wells were seeded for single and combination drug experiments, respectively. Metabolic activity of viable cells was measured on day four of treatment following addition of the MTS reagent (Promega), using an Infinite200PRO (Tecan) instrument.

### Immunoblot

Cells were pelleted by centrifugation, washed with ice-cold PBS and lysed on ice for 20 min in modified NP-40 buffer (10mM/1mM KPO4/EDTA pH 7.05, 5mM EGTA pH 7.2, 10mM MgCl2, 50mM glycerophosphate pH 7.2, 0.5% NP-40, 0.1% Brij-35) supplemented with protease inhibitor mixture (Roche), phosphatase inhibitor mixture (Roche), 1mM DTT, 1mM Va3VO4 and 1mM PMSF. Protein concentrations of whole-cell lysates were determined by BCA protein assays (Pierce), and equivalent protein amounts (20-60 μg) were electrophoresed through 4-12% Bis-Tris acrylamide gels. Proteins were transferred to 0.45 μM nitrocellulose membranes (Invitrogen) and blocked with 5% milk. Antibodies detecting pAKT(Ser473)XP, pAKT(Thr 308), AKT, pPRAS40(Thr246), PRAS40, p110α, p110β, p110γ, p85, Syk, pSyk(Tyr525/526), pZap-70(Tyr319)/Syk(Tyr352), pSrc(Tyr416), pCD19(Tyr531), CD19, pPLCγ2(Tyr759), pCD79A(Tyr182), pFoxO1(Thr24)/FoxO3a(Thr32)/FoxO4(Thr28), CD79, p-tyrosine were purchased from Cell Signaling. CD19, Syk, p110δ and actin-HRP were purchased from Santa Cruz. BCAP was purchased from R&D. Anti-rabbit IgG and anti-mouse IgG (peroxidase-conjugated) were purchased from GE Healthcare Life Sciences. Light chain-specific anti-mouse IgG, anti-rabbit IgG and anti-goat IgG (peroxidase-conjugated) were purchased from Jackson ImmunoResearch.

### Immunoprecipitation

10^7^ cells/mL were pelleted by centrifugation, washed and resuspended in PBS and lysed on ice in equal volume of 2x NP-40 lysis buffer (40 mM Tris-HCL pH8, 274mM NaCl, 2% NP-40, 4mM EDTA) supplemented with protease inhibitor mixture (Roche), phosphatase inhibitor mixture (Roche), 2mM DTT, 2mM Va3VO4 and 2mM PMSF. Protein concentrations were determined by BCA protein assay (Pierce) and were equalized among samples. Cell lysates were incubated with CD19 (Santa Cruz), BCAP (R&D) or IgG (goat, Santa Cruz) at 4°C. Sepharose beads (ThermoFisher) were washed with PBS and resuspended in 1x NP-40 lysis buffer. Beads were added to the protein lysate and incubated at 4°C with rotary agitation for 1.5h. Immunoprecipitates were centrifuged and washed with lysis buffer to remove non-specific binding. Immunoprecipitates were electrophoresed through 4-12% Bis-Tris acrylamide gels. Proteins were transferred to 0.45 μM nitrocellulose membranes (Invitrogen) and blocked with 5% milk.

### shRNA toxicity screen

shRNAs of interest were cloned into the GFP-expressing retroviral vector pRSMX-PG and cells were retrovirally transduced by spin-infection. shRNA expression was induced using doxycycline (200ng/mL) [4]. For single shRNA knockdown experiments, puromycin selection preceded doxycycline induction. For toxicity screens, puromycin selection was not performed. The fraction of GFP+, shRNA-expressing cells was measured with flow cytometry, and normalized with the day 0 fraction of uninduced GFP+ cells. Toxicity was calculated as depletion of the GFP+ cells over time.

The targeting sequences of the shRNA vectors used in this study are as follows:

p110α #1: GCACAATCCATGAACAGCATT (3′-UTR);

p110α #2: TACAGCAAGAACAGAAATAAA (3′-UTR);

Sc4 (Control): CTCTCAACCCTTTAAATCTGA.

### NF-κB reporter assays

IκBα luciferase and NF-κB-dependent transcriptional reporters were used as previously described [17, 25]. Cells were seeded in 96-well plates (2.5 × 10^5^ cells/mL) for drug treatment. Luciferase activity (Dual-Luciferase Reporter Assay System; Promega) was measured using an Infinite200PRO instrument (Tecan).

## SUPPLEMENTARY MATERIALS FIGURES


