# Supplementary material for: PI3Kδ inhibition causes feedback activation of PI3Kα in the ABC subtype of diffuse large B-cell lymphoma
Source: Oncotarget. 2017 Sep 13;8(47):81794–802. doi: 10.18632/oncotarget.20864 (PMC5669848; doi:10.18632/oncotarget.20864)
Supplement: Supplementary file 1 [file oncotarget-08-81794-s001.pdf]

PI3Kδ inhibition causes feedback activation of PI3Kα in the ABC subtype of diffuse large B-cell lymphoma

SUPPLEMENTARY MATERIALS

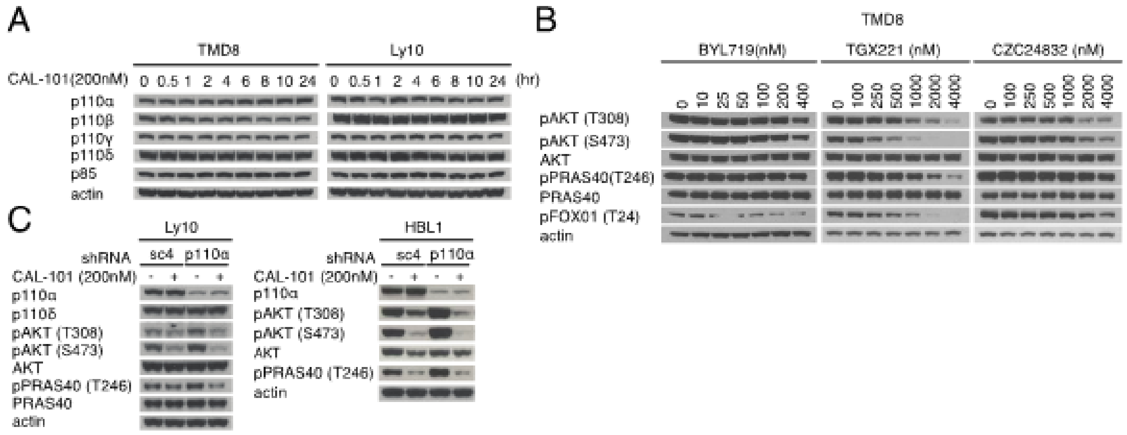

Supplementary Figure 1: Compensatory role of PI3Kα in ABC DLBCL subgroup. (A) TMD8 and LY10 were exposed over different time periods to CAL-101. Immunoblot indicates that PI3Kα expression increases over time in TMD8 but not Ly10 cells. (B) TMD8 cells were treated with different concentrations of BYL719 (PI3Kα), TGX221 (PI3Kβ) and CZC24832 (PI3Kγ). Immunoblot indicates the PI3K activity after 2hrs of drug treatment. (C) Ly10 and HBL1 cells were transduced with sc4 (control) or PI3Kα-targeted shRNA for 24hr, followed by 24hr CAL-101 treatment. Results indicate that the knock-down of PI3Kα prolongs the PI3K inhibition of CAL-101 without decreasing the baseline PI3K activity.

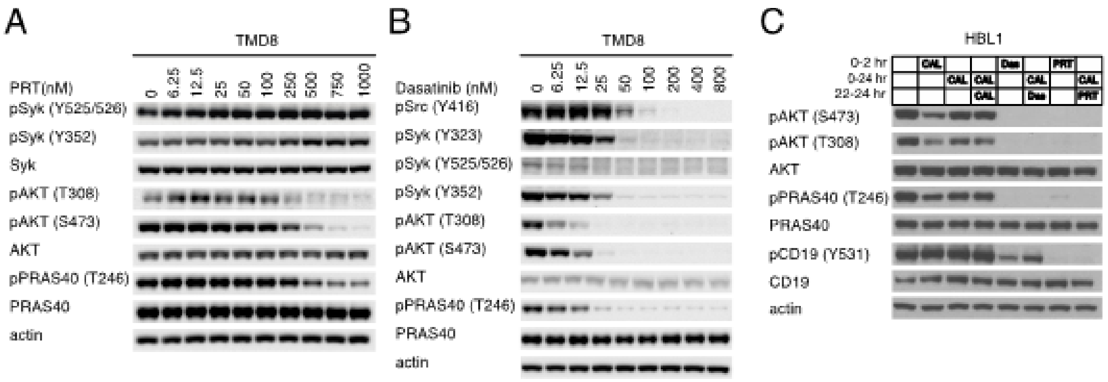

Supplementary Figure 2: Feedback activation of PI3Kα following PI3Kδ inhibition is mediated through increased BCR signaling. (A) TMD8 were treated with PRT062607 at the indicated concentrations for 2hrs. Immunoblot indicates complete inhibition of pAKT-473 and pAKT-308 at 1000nM. (B) TMD8 cells were treated with dasatinib at the indicated concentrations for 2hrs. Immunoblot indicates complete inhibition of pAKT-473 and pAKT-308 at 50nM. (C) HBL1 were treated with 200nM CAL-101, 50nM dasatinib (src inhibitor), 1000nM PRT062607 (Syk inhibitor) at the indicated time points and harvested at 2hr and 24hr. Results indicate that rebound PI3K reactivation following PI3Kδ inhibition is sensitive to Src and Syk inhibition.
